# Alterations in the steroid hormone receptor co-chaperone FKBPL are associated with male infertility: a case-control study

**DOI:** 10.1186/1477-7827-8-22

**Published:** 2010-03-08

**Authors:** Olaf Sunnotel, Laszlo Hiripi, Kevin Lagan, Jennifer R McDaid, Johanny M De León, Yasushi Miyagawa, Hannah Crowe, Soniya Kaluskar, Michael Ward, Catherine Scullion, Alan Campbell, CS Downes, David Hirst, David Barton, Edgar Mocanu, Akira Tsujimura, Marc B Cox, Tracy Robson, Colum P Walsh

**Affiliations:** 1 Transcriptional Regulation and Epigenetics, School of Biomedical Sciences, University of Ulster, Coleraine BT52 1SA, UK; 2 Border Biomedical Research Center, University of Texas at El Paso, TX 79902, USA; 3 Dept of Urology, University of Osaka Graduate School of Medicine, Suita, Osaka, Japan; 4 Cancer and Ageing Research Group, School of Biomedical Sciences, University of Ulster, Coleraine BT52 1SA, UK; 5 School of Pharmacy, Queen's University, Belfast BT9 7BL, UK; 6 National Centre for Medical Genetics Our Lady's Children's Hospital, Crumlin, Dublin, Ireland; 7 Human Assisted Reproduction Ireland, Rotunda Hospital, Dublin 1, Ireland

## Abstract

**Background:**

Male infertility is a common cause of reproductive failure in humans. In mice, targeted deletions of the genes coding for FKBP6 or FKBP52, members of the FK506 binding protein family, can result in male infertility. In the case of FKBP52, this reflects an important role in potentiating Androgen Receptor (AR) signalling in the prostate and accessory glands, but not the testis. In infertile men, no mutations of FKBP52 or FKBP6 have been found so far, but the gene for FKBP-like (FKBPL) maps to chromosome 6p21.3, an area linked to azoospermia in a group of Japanese patients.

**Methods:**

To determine whether mutations in FKBPL could contribute to the azoospermic phenotype, we examined expression in mouse and human tissues by RNA array blot, RT-PCR and immunohistochemistry and sequenced the complete gene from two azoospermic patient cohorts and matching control groups. FKBPL-AR interaction was assayed using reporter constructs in vitro.

**Results:**

FKBPL is strongly expressed in mouse testis, with expression upregulated at puberty. The protein is expressed in human testis in a pattern similar to FKBP52 and also enhanced AR transcriptional activity in reporter assays. We examined sixty patients from the Japanese patient group and found one inactivating mutation and one coding change, as well as a number of non-coding changes, all absent in fifty-six controls. A second, Irish patient cohort of thirty showed another two coding changes not present in thirty proven fertile controls.

**Conclusions:**

Our results describe the first alterations in the gene for FKBPL in azoospermic patients and indicate a potential role in AR-mediated signalling in the testis.

## Background

Genetic causes are thought to account for 10-15% of cases of severe male infertility [1]. Azoospermia is most commonly associated with microdeletions of the AZF gene on the Y chromosome [2,3], but mutations in the androgen receptor (AR) gene [4], important for sex hormone signalling, are also associated with this phenotype. Known mutations in these and other genes still only account for a fraction of azoospermia cases, suggesting that other genetic causes remain to be discovered.

Androgen insensitivity syndrome (AIS) can result in a variety of defects in the affected patient, including gynecomastia, cryptorchidism and hypospadias. Mild AIS (MAIS) on the other hand can have infertility as the only symptom and patients often show no mutations in the AR gene [5]. Defects in AR coactivators have already been implicated in one case of complete androgen resistance in humans [6] and in two sisters with partial resistance to multiple steroid hormones [7]. Homozygous deletion of the AR cochaperone FK506 binding protein 52 (FKBP52) in mice has been shown independently by two groups to result in male infertility and hypospadias with underdevelopment of the prostate and seminal vesicles [8,9]. Both groups found evidence for compromised AR activity in the knockout mice, and showed that FKBP52 potentiates AR signalling in response to androgen in prostate cell lines. Though the testes in the FKBP52 knockout mice were normal, cell type-specific AR deletions in mice have shown a requirement for the receptor in Sertoli cells during normal spermatogenesis [10,11]. This suggests that another AR cochaperone might exist in the testis which is required for optimal AR activity in these cells.

FK506 binding protein-like (FKBPL, aka DIR-1 and WISp39) was first described as a radioresponsive gene [12] and is a less well-characterised member of the FKBP family. It demonstrates binding to heat shock protein 90 (HSP90) through its conserved tetratricopeptide (TPR) repeats [13] and more recently it has been shown to interact with and stimulate the activity of the glucocorticoid receptor (GR) in human cell lines [14]. The PPI domain of FKBPL lacks crucial catalytic residues needed for the isomerase activity seen in other family members like FKBP6, which alters target protein conformation and helps regulate assembly of multiprotein complexes for clients such as ryanodine receptor and mTOR [15]. FKBP52 has one functional PPI domain, but catalytic activity may not actually be required for FKBP52-mediated regulation of AR function [16]. FKBPL maps to human chromosome 6p21.3: linkage studies in a Japanese population [17] implicate this region specifically in azoospermia (LOD score 3.5, p = 0.0005). The region contains a large number of genes, some of which were excluded by candidate studies [18]. There are also three cases of chromosomal breakpoints in this region leading to infertility in azoospermic males on the Mendelian Cytogenetics Network database, with two of these listed as azoospermic [19]. A separate report describes a family where six members carry a 6p21 translocation and show male-only (n = 3) infertility and azoospermia [20].

We examined FKBPL expression in mouse and human tissues and sequenced DNA from two human azoospermic patient cohorts, finding alterations in the gene. Furthermore, we provide evidence that FKBPL can increase transcription of AR targets in response to androgen and that the altered proteins seen in patients may be deficient in this activity.

## Methods

### Subjects

Patient samples were obtained with informed consent and approved for screening by the Ethical Approval committees of the respective institutes. The cohort of azoospermic patients from Osaka University, Japan, have been previously described and patients showing obstructive azoospermia or chromosomal abnormalities were excluded [17]: DNA from a control group of healthy Japanese males was obtained from the same source. The thirty azoospermic patient samples from Human Assisted Reproduction Ireland, Rotunda Hospital, Dublin, Ireland showed no Y chromosome microdeletions or chromosome abnormalities: the control group here were thirty proven fertile males [21]. Obstructive azoospermia was excluded for both patient groups.

### Mice

Tissues were collected from Swiss/To (Harlan, UK) mice, which were maintained in accordance with the Home Office regulations under project licence to CPW.

### Analysis of Fkbpl mRNA expression by RT-PCR

Total RNA was extracted using the RNeasy Mini Kit, including the optional DNAse treatment, following the manufacturer's instructions (Qiagen, Crawley, UK) and 1 μg used to make cDNA in a 12.5 μl mixture containing 10 mM Tris HCL (pH 8.3), 0.2 μg Oligo(dT)_15 _primer (Promega, Southampton, UK), 1.5 mM dNTPs, 1× AMV-RT buffer and 7.5 U AMV reverse transcriptase (Promega, Southampton, UK). Primers specific for *Fkbpl *(musdirf5ex CTTCCAGGCCTCAACATCAT and musdirR TCCCAGCTCGAAACAGTTCT: chr17 34781824-34782871 NCBI build 37; cDNA 756 nt; DNA 1028 nt) or β-actin (Bact1 GCTGTGCTATGTTGCTCTAGACTTC and Bact2 CTCAGTAACAGTCCGCCTAGAAGC: chr5 143665461-143666180; cDNA 500 nt; DNA 730 nt) were supplied by Invitrogen, Paisley, UK. All primers were checked for uniqueness and location using the BLAT tool available at the UCSC website [22]. PCR was performed in 25 μl containing 1× Taq buffer, 200 μM dNTPs, 0.4 μM primer, 2 U Taq (Invitrogen, Paisley, UK) and 1 μl cDNA. Initial deannealing at 94°C for 3 min was followed by 28 cycles of 45 sec at 94°C, 1 min at 61°C, and 1 min at 72°C and a final elongation for 5 min at 72°C. PCR products were separated on agarose gels and images captured using a Kodak digital camera.

### RNA expression analysis using radioactive probes

A Mouse RNA Master Blot array normalised to provide semi-quantitative data on tissue specificity and target mRNA abundance was purchased from BD Biosciences, Cowley, UK. Human and mouse *FKBPL *coding-region probes were amplified by PCR using: HumanF CTAGGCTCCTGCTGCCGGCTACTG and HumanR TCAGCAGTTGCTTTTTCCAGGTCC; musdirF GAACGAGAAGAACACCGCTC and musdirR TCCCAGCTCGAAACAGTTCT. Northern blotting, radiolabelling of cDNA and detection of mRNA were as previously described [23].

### Nucleotide sequence screening of FKBPL gene

The whole FKBPL gene was directly amplified from patient DNA using primers 5'-GGCTCCAGGGTTAGTTGTCA-3' and 5' CCCAAATCTCACAGCACA GA-3' and purified with the Wizard Gel PCR clean-up kit (Promega, Southampton, UK). Sequencing was done using primers S1 5'-AACCAGTCAGATGCCAGAGG-3', S2 5'-CCTCTGG CATCTGACTGGTT-3', S3 5'-GAACCAGGTTCAGGTCAGC-3', S4 5'-GACTAG CGAGAAGGAAGCC-3' and S5 5'-GGCTTCCTTCTCGCTAGTC-3' on an ABI Prism 3130 at the Centre for Molecular Biosciences Sequencing Facility. Patient sequences were compared with the REFSEQ entry and examined for known variations using the UCSC browser [22]. Zygosity of mutations was confirmed by sequencing TOPO-TA (Invitrogen, Paisley, UK) cloned PCR products.

### Generation of expression constructs and reporter assays

N-terminal GFP-tagged constructs were generated by cloning into pcDNA3.1/NT-TOPO-GFP (Invitrogen, Paisley, UK) using the primers fkbplF0: GAGACCCCACCA CTCAATAC and fkbplR0: TCAGCCAAACATCTTGCCC. LNCaP cells (4 × 10^4 ^per well) were seeded in 24-well plates coated with fibronectin (Invitrogen, Paisley, UK) and maintained in phenol red-free RPMI1640 with 10% charcoal-dextran stripped FBS and 10 mM HEPES at 5% CO_2 _for 24 h. Cells were then co-transfected for 6 h using lipofectamine (Invitrogen, Paisley, UK) with pPA6.1Luc reporter construct, GFP-FKBPL-WTl or a pcDNA3.1 empty vector control together with the pBIND Renilla transfection efficiency control. The transfection mix was then replaced by the RPMI media with or without 10 nM R1881 and luciferase activity assessed 24 h post-transfection using the Dual-Glo assay system (Promega, Southampton, UK).

### Western blot analysis

Whole cell protein extracts were obtained by lysis with extraction buffer (50 mM Tris-HCl, 150 mM NaCl, 5 mM EDTA,10% glycerol, 1% Igepal,1% protease inhibitor cocktail for mammalian cells (Sigma)), followed by centrifugation to remove debris. A 30 μg aliquot was fractionated on a 7.5% SDS-polyacrylamide gel (Biorad, Hemel Hempstead, UK) and transferred to a nitrocellulose membrane (GE Healthcare, Amersham, UK). Membranes were blocked for 1 h at RT with 5% preimmune serum in Tris-buffered saline with 0.2% Tween-20 (TBST) prior to incubation with a 1:800 dilution of anti-FKBPL rabbit polyclonal IgG (cat. no. 10060-1-AP, Proteintech Group, Chicago, USA) in TBST. After washing with TBST, membranes were exposed to a horseradish peroxidise (HRP) conjugated secondary antibody (sc-3837, Santa Cruz Biotechnology, Santa Cruz, USA) and signal detected using ECL reagents (GE Healthcare, Amersham, UK).

### Immunohistochemistry

Paraffin-embedded sections of a fertile human testis were obtained from ProSci Inc, Poway, California, USA. For FKBPL and FKBP52, tissue was dewaxed and rehydrated through an alcohol series prior to antigen retrieval by heating in 10 mM citrate buffer (pH 6.0) using a 600 W microwave oven (2 × 3 min). Blocking was carried out with 10% serum in PBS-Tween20 (PBST) for 1 h at RT followed by 15 min with 1% hydrogen peroxide to remove endogenous peroxidase activity. Sections were washed twice with PBST then incubated overnight with 1:100 rabbit anti-human FKBPL (ProteinTech Group Inc, Chicago) or 1:250 dilution of anti-FKBP52 (cat#10011442, Cayman Chemical Company, Ann Arbor, Michigan, USA) at 4°C, followed by 1 h incubation with 1:2000 goat anti-rabbit IgG-HRP. Immunostaining was carried out with DAB Substrate Plus kit (Zymed, South San Francisco, USA), before counterstaining with hematoxylin (Sigma, Welwyn Garden City UK), dehydrating and mounting in Mowiol (Calbiochem, Nottingham, UK). For AR, immunostaining was essentially as described previously for this antibody (PG21, Millipore, Billerica, MA) [24], with a Vectastain ABC kit (Vector, Peterborough, UK) used for signal amplification and the Impact DAB kit (Vector) used for staining.

### Statistical analysis

To examine differences in mutation frequencies between patient and control groups, Chi-squared values were calculated from a contingency table (Yate's method) and used to calculate significance values (*p*) by Fisher's exact method in the PRISM application (Graphpad, San Diego, USA).

## Results

### Structure and expression

**Figure 1:**
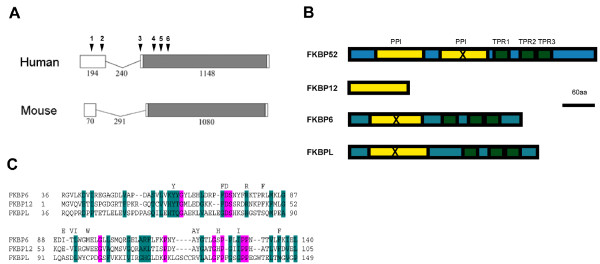
**FKBPL structure and conservation**. **A**. Structure of the human and mouse genes. The gene consists of two exons, joined by a short intron. The open reading frame is in grey, lengths in base pairs are indicated. The positions of the gene alterations seen in patients and listed in Table 1 are indicated above the human sequence. **B**. Protein structure. Peptidyl-prolyl *cis-trans *isomerase (PPI) domains are shown in yellow, tetratricopeptide repeats (TPR) in green. FKBP12 has a PPI domain but contains no TPR. FKBP52 and FKBP51 contain a duplication of the PPI domain, but the C-terminal copy is inactive (X). FKBP6 and FKBPL have N-terminal regions with some homology to the PPI.C. Alignment of the PPI domains from FKBP6, FKBP12 and FKBPL by CLUSTALW: residues conserved in PPI with good enzymatic activity are indicated above the alignment but can be seen to be poorly conserved in FKBPL.

*FKBPL *is encoded by a small two-exon gene (Fig. 1A). The protein contains a peptidyl-prolyl *cis-trans *isomerase (PPI) domain (Fig. 1B) and a region containing tetratricopeptide repeats (TPR), like other members of the FKBP family of proteins, which act as cochaperones for steroid receptor complexes. FKBPL shows low homology over the PPI domain and lacks critical residues that have been shown to be required for enzymatic activity (Fig. 1C), but is relatively well conserved at the TPR, which have been shown to be functional in binding HSP90 [13]. The protein is highly conserved across several mammalian species, indicative of an important function.

**Figure 2:**
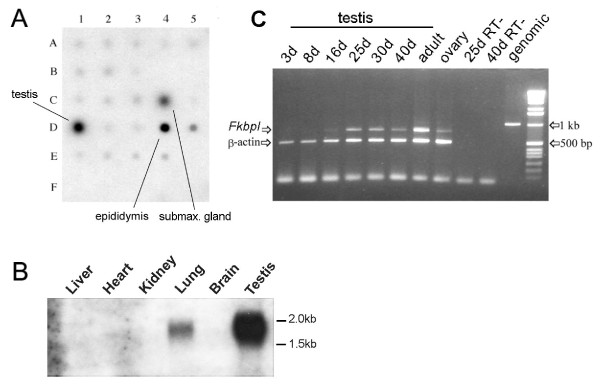
**Fkbpl transcription in mouse**. **A**. A Multiple Tissue Array normalised polyA+ RNA blot was hybridised to a radiolabelled *Fkbpl *cDNA. The locations of testis, epididymis and submaxillary gland RNA are indicated. Top row: brain (A1), eye (A2), liver (A3), lung (A4), kidney (A5); Second row: heart (B1), skeletal muscle (B2), smooth muscle (B3), pancreas; Third row: (C1), thyroid (C2), thymus (C3), submaxillary gland (C4), spleen (C5); Fourth row: testis (D1), ovary (D2), prostate (D3), epididymus (D4), uterus (D5); Fifth row: embryo 7 days (E1), embryo 11 days (E2), embryo 15 days (E3) and embryo 17 days (E4); Last row: negative controls (F1-4). Positions not listed (e.g. B4) are blank. **B**. Northern blotting. Total RNA (20 ug) was extracted from the tissues indicated and fractionated on a 1% gel containing formamide before transferring to nitrocellulose and hybridising to the cDNA probe used in A. The positions of relevant size markers are indicated at right. The experiment was repeated twice. **C**. RT-PCR of total RNA isolated from testis at different days postnatally. The primers span the intron, allowing any contaminating genomic product to be easily distinguished (right); β-actin is used as an internal positive control: RT- are negative controls. The experiment was repeated twice.

**Figure 3:**
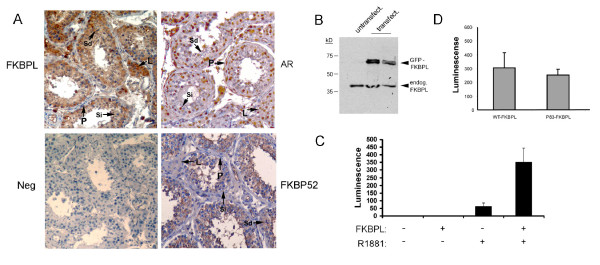
**FKBPL expression in human testis**. **A**. Immunohistochemical staining of human testis sections with the indicated antibodies. AR shows nuclear staining in Sertoli (Si), Leydig (L) and peritubular myoid (P) cells, but is negative elsewhere, including spermatids (Sd). FKBP52 is cytoplasmic, staining Sertoli and spermatogenic cells, but shows weak or absent staining in Leydig and myoid cells. FKBPL is also cytoplasmic, matching FKBP52 in most respects except for strongly staining the Leydig cells. Preimmune serum for FKBPL (shown) and other antibodies was used as a negative control (neg.). Experiments were repeated three times. Staining for FKBPL and FKBP52 was almost identical in rat testis (not shown). **B**. Western blot of total protein from untransfected HT29 cells (untransfect.) or transfected with a GFP-FKBPL fusion protein (transfect.). The size of the endogenous protein (endog.) is indicated. The experiment was repeated three times. **C**. LNCaP cells were transfected with a construct (pPA) containing luciferase driven by the prostate specific antigen transcriptional regulatory elements either with or without FKBPL as shown. The indicated cultures were additionally exposed to 1 nM of AR ligand (R1881). Luciferase activity was measured using a fluorimeter and normalised to an internal control. The experiment was carried out more than three times with similar results. **D**. LnCaP cells were transfected with pPA and FKBPL from normal controls (WT) or from the mutated allele seen in patient 83 (p83- listed as #6 in Table 1) and exposed to R1881. The results shown represent the median of three experiments.

Screening of a normalised Mouse RNA Master Blot array showed very high levels of expression in testis and epididymis (Fig. 2A), high levels in submaxillary gland and low levels in all other tissues: this was confirmed by northern blotting (Fig. 2B). RT-PCR of testis mRNA showed that transcription of the gene is turned on during sexual maturation in the male mouse at puberty (Fig. 2C). Expression in humans was assessed using a similar array which contained 75 tissue-specific polyA+ RNAs: transcription was more widespread, but was again strongest in testis (data not shown). An antibody has recently been raised to FKBPL. We carried out immunostaining on human testis sections to see if FKBPL is expressed here (Fig. 3A). Staining was found in most cells of the tubule, including the Sertoli cells, which can be distinguished by an ovoid nucleus with a single prominent nucleolus [25]. There was no staining in the peritubular myoid cells or blood vessels but the interstitial Leydig cells were prominently stained. Staining of the same sections with an antibody to AR showed nuclear staining of Sertoli, Leydig and peritubular myoid cells but no signal was visible in the germ cells such as spermatids (Fig. 3A), as previously reported [24]. Staining for FKBP52 gave a similar pattern to FKBPL but with very weak or absent staining of the Leydig cells (Fig. 3A). Staining carried out with preimmune serum as a negative control gave no background. To confirm the specificity of the FKBPL antibody we carried out western blotting on human cell lines carrying a GFP-tagged version of the human protein (Fig. 3B). The antibody detected both endogenous and transfected FKBPL, with little or no background.

### Evidence for involvement in human infertility

We found that *FKBPL *in humans maps to a region linked to azoospermia in a Japanese population (LOD score 3.5, p = 0.0005) [17]. We examined 60 of the patient samples used in that study and 56 controls from the same population and looked for mutations in the FKBPL gene by direct sequencing, which we numbered in order from the 5' end of the gene (Table 1 and Fig. 1A). This identified two patients carrying heterozygous mutations in the gene in the azoospermic group, one a mutation in the canonical splice acceptor site (C**A**G/G -> C**G**G/G) for the only coding exon, shown previously to give loss of function [26] (mutation 3, Table 1), and the other an insertion which is predicted to alter a binding pocket (mutation 6, Table 1). These mutations were confirmed by cloning PCR products and sequencing individual clones and by sequencing of blinded samples at a second laboratory. Neither mutation was present in our control group. We also examined a cohort of 30 patients from an Irish infertile male population where Y chromosome microdeletions and chromosomal abnormalities have been excluded and a matched control group of 30 fertile Irish males. Two of the Irish patient samples had single amino acid changes in the coding region (mutations 4 and 5, Table 1) which were not found in the control group. The Japanese and Irish patients also showed significantly different frequencies of synonymous mutations at two sites compared to controls (mutations 1 and 2, Table 1), suggesting that these alterations may be functionally significant or tightly linked to as-yet unidentified alterations elsewhere.

**Table 1:** Sequence differences between azoospermic patients and matched controls^a^

Mutation	Type	Alteration	**Location**^**b**^	Frequency
1	Base change	Synonymous	32205968	0 Japanese patients,^c^ 12 controls
2	Base change	Synonymous	32205869	10 Japanese patients,^d^ 0 controls
2^f^	Base change	Synonymous	(as above)	8 Irish patients,^e^ 1 control
3	Splice site	Loss of function	32205611	1 Japanese patient, 0 controls
4	Base change	Asn=>Ser	32205453	1 Irish patient, 0 controls
5	Base change	Thr=>Arg	32205391	1 Irish patient, 0 controls
6	Insertion	4AA duplication	32205307	1 Japanese patient, 0 controls

^a^Group sizes: Japanese patients (n = 60) and Japanese controls (n = 56); Irish patients (n = 30) and Irish controls (n = 30).

^b^Location given with respect to the International Human Genome Sequencing Consortium March 2006 human reference sequence (NCBI Build 36.1).

^c^Probability *p *<*0.001*; ^d ^*p *= *0.0014*, ^e ^*p *= *0.026 *by Fisher's exact test, see also methods

^f^Listed twice, as the frequencies and associated probabilities differ in the two populations

### Possible function of FKBPL in testis

The azoospermic and infertile nature of our patient group, combined with the expression pattern of the protein, suggested that FKBPL, like FKBP52, might be a co-chaperone for Androgen Receptor in the testis. To check for AR interaction, we used the androgen-responsive prostate cancer cell LNCaP which have high levels of AR. These were transfected using a reporter construct (pPA) containing luciferase downstream of the prostate specific antigen (PSA) transcriptional regulatory elements [9] (kind gift of Dr. J.-T. Liu). Results are shown in Figure 3C: FKBPL enhanced AR activity on the PSA reporter specifically in response to ligand (R1881). These results show that FKBPL enhances transcriptional activation by AR of a major target gene. We confirmed this effect in mouse fibroblast cells co-transfected with FKBPL and AR in the presence of the reporter (not shown). In addition, we cloned the allele with the small insertion (mutation 6, Table 1) seen in Japanese patient 83 (p83). Results with this construct were however variable and while some experiments showed a marked decrease in enhancement of AR activity, on the whole activity of the PSA reporter in the presence of p83 protein was close to that seen with the wild-type protein (Figure 3D: a result representative of the median of the experiments is shown).

## Discussion

We describe here a number of alterations in the FKBPL gene which are found only in azoospermic males and are absent in matched controls. Four patients from two ethnic groups show different coding changes: these include mutation of the splice acceptor site for the only coding exon, a 4aa insertion in a conserved PPI binding pocket and two amino acid changes. In keeping with a potentially important role in male reproduction, the gene shows strong tissue-specific expression in the mouse testis, with transcription being initiated with the onset of sexual maturation. The human homologue is likewise strongly expressed in the cells of the testis. The protein produced is a member of a steroid hormone receptor co-chaperone family and we found the expression of FKBPL in the testis to be very similar to that of FKBP52, an androgen receptor regulator.

Both FKBP52 and FKBP6, another family member, have been implicated in male sexual development in mice, but in human case:control studies no mutations were found in either gene in azoospermic or hypospadic males [27-29]. FKBP6 has been reported to be a structural component of the synaptonemal complex [30]: we could confirm from reprobing our array blot that *Fkbp6 *was expressed in testis only, and not epididymis and submaxillary gland where *Fkbpl *levels were high (data not shown). Expression in submaxillary gland is characteristic of steroid hormone signalling components [31]. Like *FKBP52 *[8], *FKBPL *appears to be expressed more widely in testis, including in the Sertoli and Leydig cells, than *FKBP6*, which is confined to the spermatogonial stem cells only [30].

Sertoli cells are AR-producing cells located inside the testis tubules where they play a crucial role in nurturing and supporting the development of the spermatogonia and act as a blood-testis barrier, as well as producing anti-Müllerian hormone [32]. Cell type-specific knockout of AR in Sertoli cells leads to azoospermia in mice [10,11]. FKBP52 has been shown by two groups to act as a cochaperone for AR and to boost AR transcriptional activity in response to androgen [8,9]: homozygous deletion in mice had no effect on the testis, but prostate and other secondary sexual organs expressing FKBP52 were reduced or absent. This suggests that another co-chaperone for AR might exist in the testis and FKBPL is strongly expressed in Sertoli cells. It is notable that Leydig cells also show strong AR and FKBPL staining (Fig. 3A and [24]) but show weak signal for FKBP52 (Fig. 3A and [8]). Our data show that transfection of FKBPL into the androgen-responsive human LNCaP cell line, or into mouse fibroblasts with AR, increases signalling through the receptor in response to ligand. This is consistent with a role similar to that of FKBP52, except in the testis. The fact that FKBPL can interact with GR and probably p53 in some tissues [13] also matches the multi-client protein behaviour of FKBP52, as shown by several laboratories [8,9,33] and with data from the Pratt and Sanchez groups [34,35] which suggest that the co-chaperone:client protein interaction is tissue-dependent.

The type of splice acceptor mutation in patient 25 has been shown before to result in loss of function [26] and so is predicted to prevent FKBPL production completely from this allele. If FKBPL forms part of a multimeric assembly, like other cochaperones, loss of this component might cause destabilisation of the whole complex. The insertion mutation in patient 83 is predicted to alter a binding pocket in the PPI domain of the FKBPL protein, based on the crystal structure of FKBP52. In our reporter assay, the mutated protein gave variable results but the overall trend was for a small though not statistically significant decrease in luciferase levels. This decrease in AR-mediated transcription may be more marked under physiological conditions in testis cells. The domain is highly-conserved in mammals and McKeen et al have recently shown that it is important for interaction with dynamitin and subsequent nuclear translocation of the steroid hormone:chaperone complex [14], like the PPI domain of FKBP52 [33], so the mutation also might affect this, or some other aspect of FKBPL function which is not currently well-understood.

The frequency of alterations in FKBPL in the azoospermic populations is not inconsistent with the frequencies seen for other human genes implicated in infertility, such as Y chromosome microdeletions (2-20% of infertile males [3]) or in particular the other autosomal genes such as SYCP3 (heterozygous mutations in 2/19 infertile males [36]); USP9 (17/576 patients or 3% [37]) and Protamine 1 (heterozygous mutations in 1-10% of patients [38,39]. A large number of genes contribute to normal fertility, so it is expected that the individual contributions of any one gene will be low. The heterozygous nature of the mutations uncovered so far, a feature of the other genes implicated in human infertility listed above and consistent with a recent screen [30], could indicate haploinsufficiency, or that the mutation on the other chromosome is as yet undetected. It is also of note that the FKBP6 gene in humans has been reported to be monoallelically expressed [29].

## Conclusions

Linkage analysis and clustering of chromosomal breakpoints in infertile males both implicate the region containing the FKBPL gene in azoospermia. Here we show that the protein, like its paralogs, can act as an AR cochaperone and is expressed at high levels specifically in mature testis. Azoospermic patient groups from two populations show mutations in the gene not present in matched controls. Overall, these results indicate that mutations in FKBPL are associated or partially responsible for infertility in males and shed light on its expression and role in the normal testis.

## Competing interests

The authors hold a patent for the use of FKBPL in screening for mutations in infertile males (WO 2009/077158).

## Authors' contributions

OS carried out GFP fusions; LH carried out array blots and RT-PCR; OS, LH, JMcD, KL, HC, SK, MW, CS, AC and CPW carried out sequencing; HC and LH carried out northern blots; KL and OS carried out immunohistochemistry; OS and JMDL carried out reporter assays; YM, EM, DB and AT provided clinical samples; CPW designed the experiments and carried out sequence comparisons; CSD, DH, MBC, TR, DB, AT and CPW wrote the manuscript. All Authors have read and approve the final manuscript.
